# ICTV Virus Taxonomy Profile: Fusariviridae 2024

**DOI:** 10.1099/jgv.0.001973

**Published:** 2024-04-15

**Authors:** Sotaro Chiba, Nobuhiro Suzuki, Leonardo Velasco, María A. Ayllón, Shin-Yi Lee-Marzano, Liying Sun, Sead Sabanadzovic, Massimo Turina

**Affiliations:** 1 Nagoya University, Furo-cho, Chikusa-ku, Nagoya 464-0861, Japan; 2 Okayama University, Kurashiki 710-0046, Japan; 3 Instituto Andaluz de Investigación y Formación Agraria, Centro de Málaga, 29140 Malaga, Spain; 4 Universidad Politécnica de Madrid, Madrid 28040, Spain; 5 United States Department of Agriculture, Toledo, OH 43606, USA; 6 Northwest A&F University, Taicheng Road 3#, Yangling, Shaanxi, 712100, PR China; 7 Mississippi State University, Mississippi State, MS 39762, USA; 8 Institute for Sustainable Plant Protection-CNR, Torino 10135, Italy

**Keywords:** *Fusariviridae*, ICTV Report, Taxonomy

## Abstract

*Fusariviridae* is a family of mono-segmented, positive-sense RNA viruses with genome sizes of 5.9–10.7 kb. Most genomic RNAs are bicistronic, but exceptions have up to four predicted ORFs. In bicistronic genomes, the 5′-proximal ORF codes for a single protein with both RNA-directed RNA polymerase (RdRP) and RNA helicase (Hel) domains; little is known about the protein encoded by the second ORF. Fusarivirids do not appear to form virions. This is a summary of the International Committee on Taxonomy of Viruses (ICTV) Report on the family *Fusariviridae,* which is available at ictv.global/report/fusariviridae.

## Virion

No true virions are associated with fusarivirids (Table 1).

**Table 1.** Characteristics of members of the family *Fusariviridae*

Example:	Fusarium graminearum dsRNA mycovirus 1 (AY533037), species *Alphafusarivirus fusarii*, genus *Alphafusarivirus*
Virion	Thought to be capsidless since no virions can be purified on a sucrose density gradient
Genome	5.9–10.7 kb mono-segmented, positive-sense RNA
Replication	Unknown
Translation	For Fusarium graminearum dsRNA mycovirus 1, expression of downstream ORFs is from subgenomic RNAs. There is no evidence for such molecules for Rosellinia necatrix fusarivirus 1
Host range	Experimentally confirmed hosts are members of the kingdom Fungi and in one case a confirmed stramenopiles oomycetes host
Taxonomy	Realm *Riboviria*, kingdom *Orthornavirae*, phylum *Pisuviricota*, class *Duploviricetes*, order *Durnavirales*: several genera and >30 species

## Genome

**Fig. 1.**
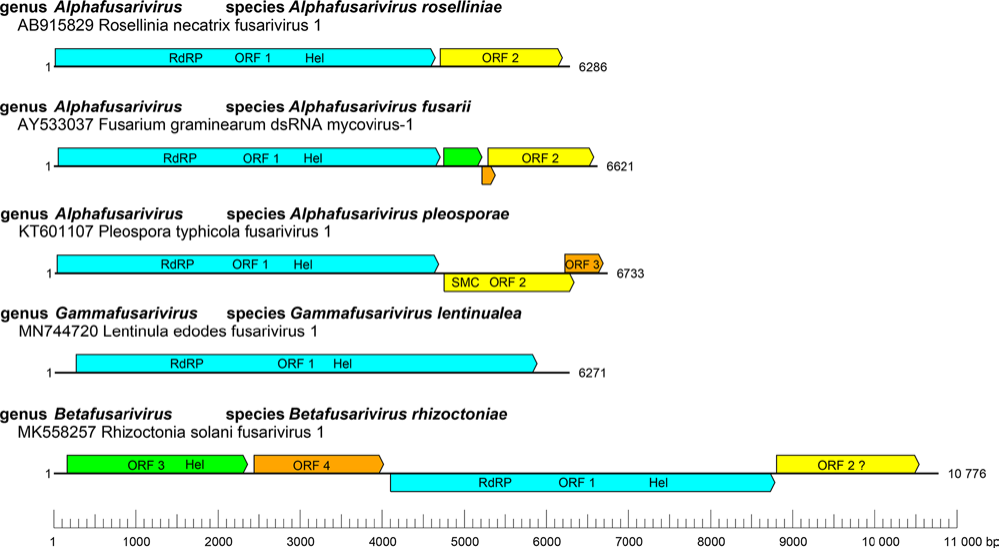
Genome organisation of representative fusariviruses. RdRP, RNA-directed RNA polymerase domain, Hel, Helicase domain, SMC, structural maintenance of chromosomes domain.

Fusarivirids can be monocistronic (Lentinula edodes fusarivirus 1) [1], bicistronic (Rosellinia necatrix fusarivirus 1) [2], tricistronic (Pleospora tiphycola fusarivirus 1) [3] or quadricistronic (Rhizoctonia solani fusarivirus 1) [4] (Fig. 1). The largest ORF always codes for a single protein with both RNA-directed RNA polymerase (RdRP) and RNA helicase (Hel) domains. The second ORF encodes a protein that contains a motif from a structural maintenance of chromosomes (SMC) protein (PDB entry code: 6YUF) [5,6]. Accessory ORFs are often genus-specific. In the largest quadricistronic fusarivirids a second helicase domain is also present [7].

## Replication

During replication, the abundant replicative form of dsRNA accumulates intracellularly.

## Pathogenicity

A comparison of colony morphology between virus-free and virus-infected isogenic strains suggests that infections of *Rosellinia necatrix* with Rosellinia necatrix fusarivirus 1 is asymptomatic and does not cause hypovirulence on apple rootstocks [2]. Lack of symptoms has also been observed for Sclerotinia sclerotiorum fusarivirus 1 [8]. Fusarium graminearum virus 1 strain SD4 causes hypovirulence and eliminates deoxynivalenol synthesis in *Fusarium graminearum* [9].

## Taxonomy

**Fig. 2.**
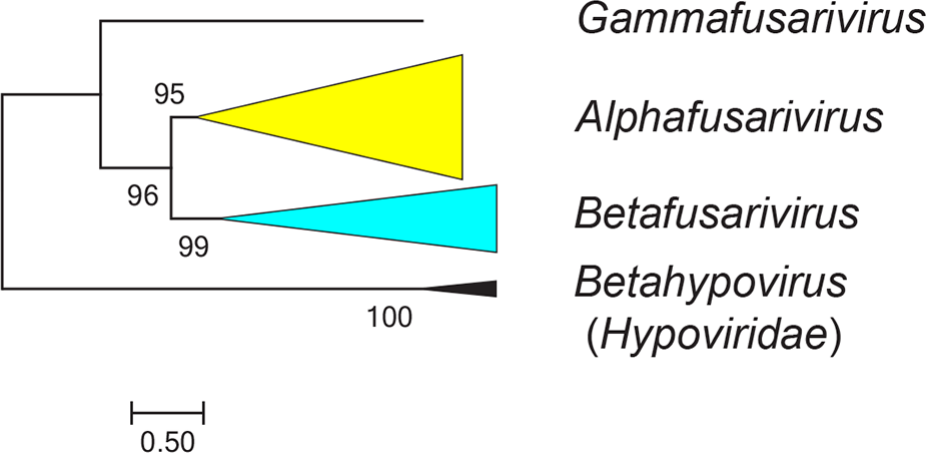
Phylogenetic analysis of fusarivirus RdRP protein sequences. Numbers at nodes indicated percentage bootstrap support. For details of viruses and methods see full *Fusariviridae* ICTV Report.

Current taxonomy: ictv.global/taxonomy. The family *Fusariviridae* (Fig. 2) is included in the pisuviricot class *Duploviricetes*, order *Durnavirales*, together with a number of families mostly with dsRNA genomes, with the exception of the most closely related family (*Hypoviridae*), members of which have a ssRNA genome [2,10].

## Resources

Full ICTV Report on the family *Fusariviridae*: http://www.ictv.global/report/fusariviridae.

## Abbreviations

Hel: RNA helicase
RdRP: RNA-directed RNA polymerase
SMC: a structural maintenance of chromosomes protein
